# Secure Messaging and Telephone Use for Clinician-to-Clinician Communication

**DOI:** 10.1001/jamanetworkopen.2024.17781

**Published:** 2024-06-20

**Authors:** Sunny S. Lou, Daphne Lew, Laura R. Baratta, Elise Eiden, Christine A. Sinsky, Thomas Kannampallil

**Affiliations:** 1 Department of Anesthesiology, Washington University School of Medicine in St Louis, St Louis, Missouri; 2 Institute for Informatics, Washington University School of Medicine in St Louis, St Louis, Missouri; 3 Center for Biostatistics and Data Science, Washington University School of Medicine in St Louis, St Louis, Missouri; 4 Division of Biology and Biomedical Sciences, Washington University School of Medicine in St Louis, St Louis, Missouri; 5 American Medical Association, Chicago, Illinois

## Abstract

This cohort study investigates the association of use of text-based secured messaging with telephone use among resident physicians.

## Introduction

Communication between clinicians is essential for effective patient care. Historically, the telephone has been the primary method for clinician-to-clinician communication. However, with the advent of mobile devices and electronic health record (EHR) systems, text-based secure messaging platforms have grown in use rapidly, with studies suggesting a doubling in use over the past 5 years.^1,2^ Despite widespread adoption, the association of secure messaging with clinician communication practices remains poorly understood. Many have hypothesized that secure messaging may replace highly interruptive telephone calls, potentially leading to more efficient workflows^3^; however, secure messaging may also be used alongside telephone to increase the overall burden of communication.^4^ This study assesses the association between secure messaging and telephone use.

## Methods

The Washington University Institutional Review Board approved this cohort study with a waiver of informed consent because it was a retrospective study with minimal risk to participants. The study is reported using the STROBE guidelines. All resident physicians at Barnes-Jewish Hospital between August 1, 2022, and January 31, 2023, were included except 3 for whom sex was missing. Each resident was issued a smartphone at residency enrollment with instructions to use it for workplace communication; the same phone was used throughout residency.

Metadata on monthly telephone minutes were extracted from mobile device billing data. Metadata on EHR-integrated secure messaging use (Secure Chat, Epic Systems; implemented in 2019) were extracted from the EHR data warehouse.^2^ Monthly secure messaging volume was measured as the sum of all messages sent and received. Data were collected for 6 months and analyzed at the person-month level; 347 person-months with 0 telephone minutes and 0 secure messages were excluded.

To assess the association between secure messaging volume and telephone minutes, a mixed-effects linear regression model was created. Secure messaging volume and sex were used as fixed effects, and resident and clinical rotation were used as random effects to adjust for individual preferences and differences in communication need between rotations. The most frequent EHR login context for each month was used to determine clinical rotation assignments. A 2-sided *P* < .05 was used for statistical significance. Analysis was conducted using SAS statistical software version 9.4 (SAS Institute).

## Results

A total of 1057 resident physicians (594 male [56.2%]) and 5995 person-months were included, representing 18 specialties and 231 clinical rotations (Table 1). Each resident sent or received a median (IQR) of 148 (25-561) secure messages and used a median (IQR) of 166 (21-380) telephone minutes per month. In multivariable analysis, moving from the 25th to 75th percentile (from 25 to 561 messages/mo) in monthly secure messaging volume was associated with an increase in monthly telephone call minutes of 73 minutes (95% CI, 66-80 minutes; *P* < .001) (Table 2).

**Table 1.** Study Cohort Characteristics and Associated Median Messaging and Telephone Volume

Variable	No. (%)	Median (IQR)	
		Monthly secure messaging volume	Monthly telephone minutes
**Clinician-level characteristics (N = 1057)**			
Sex			
Male	594 (56.2)	127 (21-501)	151 (11-358)
Female	463 (43.8)	191 (33-637)	186 (37-407)
Specialty			
Internal medicine	343 (32.5)	286 (66-840)	164 (40-350)
General surgery	150 (14.2)	142 (37-420)	307 (90-547)
Radiology	94 (8.9)	25 (9-73)	0 (0-27)
Anesthesiology	79 (7.5)	19 (6-70)	211 (47-303)
Emergency medicine	59 (5.6)	590 (334-839)	274 (55-423)
Psychiatry	49 (4.6)	203 (65-446)	129 (58-251)
Orthopedic surgery	45 (4.3)	1370 (306-2184)	418 (195-618)
Pathology	44 (4.2)	0 (0-9)	18 (4-47)
Neurology	40 (3.8)	319 (82-675)	293 (87-516)
Obstetrics and gynecology	39 (3.7)	301 (142-626)	76 (21-167)
Dermatology	21 (2.0)	8 (3-22)	0 (0-22)
Neurosurgery	19 (1.8)	359 (19-705)	1203 (296-1703)
Ophthalmology	17 (1.6)	49 (16-182)	299 (65-592)
Radiation oncology	16 (1.5)	31 (2-96)	215 (59-378)
Urology	15 (1.4)	112 (16-349)	369 (169-567)
Physical medicine and rehabilitation	12 (1.1)	6 (0-19)	98 (18-238)
Otolaryngology	11 (1.0)	90 (40-303)	321 (194-573)
Plastic surgery	4 (0.4)	28 (13-67)	266 (119-361)
**Top ** **20 clinical rotations among included person-mo (n = 5995)**			
General surgery	644 (10.7)	129 (35-402)	353 (165-578)
Radiology	498 (8.3)	24 (9-67)	0 (0-26)
Medicine clinic	457 (7.6)	239 (90-521)	158 (66-315)
Medicine inpatient	418 (7.0)	1063 (530-1582)	263 (153-420)
Emergency department	304 (5.1)	610 (336-847)	284 (58-428)
Anesthesiology	302 (5.0)	18 (6-61)	230 (66-315)
Pathology	221 (3.7)	0 (0-9)	18 (4-47)
Critical care	216 (3.6)	228 (34-531)	86 (5-225)
Obstetrics and gynecology	192 (3.2)	309 (146-624)	81 (24-163)
Orthopedic surgery	168 (2.8)	1879 (579-2208)	456 (253-653)
Psychiatry inpatient	113 (1.9)	406 (108-951)	221 (124-318)
Psychiatry clinic	110 (1.8)	187 (44-236)	83 (42-148)
Cardiology consult	110 (1.8)	222 (58-669)	203 (84-482)
Neurosurgery clinic	97 (1.6)	343 (11-705)	1290 (333-1734)
Neurology inpatient	90 (1.5)	408 (227-644)	391 (199-515)
Radiation oncology clinic	88 (1.5)	26 (2-85)	230 (52-387)
Neurology clinic	80 (1.3)	388 (73-870)	385 (160-672)
Infectious disease	73 (1.2)	262 (85-1010)	29 (0-112)
Ophthalmology clinic	71 (1.2)	50 (18-180)	382 (106-665)
Pulmonology clinic	70 (1.2)	25 (6-120)	58 (4-125)

**Table 2.** Association Between Monthly Secure Messaging Volume and Telephone Minutes^a^

Fixed effect	Parameter estimate (95% CI), min^b^^,^^c^	*P* value
Secure message volume (25th to 75th percentile)	73 (66 to 80)	<.001
Sex (female vs male)	16 (−12 to 44)	.26

^a^ Adjusted for sex, repeated measures among individuals, and clinical rotation as a proxy for clinical work setting and communication needs.

^b^ The parameter estimate for secure messaging volume has been scaled to illustrate the effect size of moving from the 25th to 75th percentile (from 25 to 561 messages/mo) in secure messaging volume.

^c^ Among random effects, the intraclass correlation coefficient was 0.526 for person and 0.139 for clinical rotation.

## Discussion

This cohort study found that after adjusting for sex, individual preferences, and clinical work setting, months with increased secure messaging use also had increased telephone use. This result is consistent with prior research showing that the use of new workplace communication channels may not mitigate the use of existing channels,^5^ highlighting the possibility that secure messaging may be associated with worsened communication burden and interruptions when not used thoughtfully. Although secure messaging has potential advantages, further research is needed to understand clinician communication preferences and the best strategies for secure messaging use to minimize burden.

This study has limitations. The association between secure messaging and telephone use is confounded by certain clinical settings having higher communication needs; we adjusted for this confounder using clinical rotation as a proxy. However, clinical rotation boundaries were not necessarily aligned with the calendar months of analysis. The possibility of residual confounding may exist. Not all telephone and secure messaging use may have been for patient care; similarly, residents may have sometimes used telephones other than the one issued to them. This was a single-center study of resident physicians, and results may not generalize to other settings or clinician groups.

## Notes

Supplement.
Data Sharing Statement
