# Supplementary material for: Secure Messaging and Telephone Use for Clinician-to-Clinician Communication
Source: JAMA Netw Open. 2024 Jun 20;7(6):e2417781. doi: 10.1001/jamanetworkopen.2024.17781 (PMC11190794; doi:10.1001/jamanetworkopen.2024.17781)
Supplement: Supplement. — Data Sharing Statement [file jamanetwopen-e2417781-s001.pdf]

Data Sharing Statement

Lou. Secure Messaging and Telephone Use for Clinician-to-Clinician Communication. *JAMA Netw Open*. Published online June 20, 2024. doi:10.1001/jamanetworkopen.2024.17781

Data
Data available: No
